# A System-Wide Investigation of the Dynamics of Wnt Signaling Reveals Novel Phases of Transcriptional Regulation

**DOI:** 10.1371/journal.pone.0010024

**Published:** 2010-04-07

**Authors:** Taranjit S. Gujral, Gavin MacBeath

**Affiliations:** Department of Chemistry & Chemical Biology, Harvard University, Cambridge, Massachusetts, United States of America; Center for Genomic Regulation, Spain

## Abstract

Aberrant Wnt signaling has been implicated in a wide variety of cancers and many components of the Wnt signaling network have now been identified. Much less is known, however, about how these proteins are coordinately regulated. Here, a broad, quantitative, and dynamic study of Wnt3a-mediated stimulation of HEK 293 cells revealed two phases of transcriptional regulation: an early phase in which signaling antagonists were downregulated, providing positive feedback, and a later phase in which many of these same antagonists were upregulated, attenuating signaling. The dynamic expression profiles of several response genes, including *MYC* and *CTBP1*, correlated significantly with proliferation and migration (*P*<0.05). Additionally, their levels tracked with the tumorigenicity of colon cancer cell lines and they were significantly overexpressed in colorectal adenocarcinomas (*P*<0.05). Our data highlight CtBP1 as a transcription factor that contributes to positive feedback during the early phases of Wnt signaling and serves as a novel marker for colorectal cancer progression.

## Introduction

Wnt proteins constitute a family of highly conserved growth factors [1] that mediate a wide range of biological processes, including proliferation, migration, and differentiation. They also control embryonic patterning and cell-fate decisions during early development [2], [3]. Aberrant activation of Wnt signaling has been implicated in a variety of human developmental disorders [4] and in malignancies of the colon, skin, brain, plasma, and prostate [5]. Most notably, activating mutations in components of the canonical Wnt signaling pathway are observed in >90% of colorectal cancers [6], and colorectal cancer is currently the second leading cause of cancer-related deaths in the United States [7].

The canonical Wnt signaling pathway is activated when a Wnt ligand binds to the transmembrane receptors frizzled (Fzd) and low-density lipoprotein receptor-related protein (LRP) [8], [9]. Ligand binding induces formation of a multiprotein complex that includes Dishevelled-1 (Dvl), Fzd, and Axin. Recruitment of Axin to this complex destabilizes the β-catenin degradation complex, allowing β-catenin to accumulate in the cytoplasm and translocate to the nucleus. Nuclear β-catenin activates transcription factors of the T-cell factor/lymphoid enhancing factor (TCF/LEF) family by displacing Groucho proteins and recruiting an array of co-activator proteins such as BCL9/PYG and CBP [8], [9]. This induces expression of *MYC* (c-Myc), *JUN* (c-Jun), *CCND1* (Cyclin D1), and a variety of other response genes involved in growth, differentiation, cell cycle progression, migration, and survival [10].

To date, numerous components of the Wnt network, from the plasma membrane to the cell nucleus, have been identified [8]. In addition to the canonical pathway described above, other signaling pathways are also activated by Wnt ligands [4] and a more holistic understanding of the overall topology of the network is beginning to emerge. To date, however, a system-wide and quantitative analysis of the dynamics of Wnt signaling has not been reported and it remains unclear how positive and negative regulators of the network are coordinately regulated.

To investigate how Wnt signaling proteins interrelate to control normal cellular functions such as proliferation and migration, we used serum-starved human embryonic kidney (HEK) 293 cells to follow the transcript levels of virtually every protein that has been implicated in Wnt signaling over a 24-h time course of stimulation with Wnt3a. Using self-organizing maps, we identified clusters of genes that exhibit similar expression dynamics and uncovered previously unrecognized positive and negative feedback loops. We also identified *CTBP1* as an early response gene with a dynamic expression profile that correlates with proliferation and migration. We found that CtBP1 drives signaling by upregulating β-catenin and serves as a novel marker of tumor progression in colorectal adenocarcinomas.

## Results and Discussion

Although the general topology of the Wnt network is reasonably well understood, most investigations of Wnt signaling have focused on one or a few components and have largely ignored signaling dynamics. To gain a more holistic view of how information flows through this network, we used HEK 293 cells as an *in vitro* model of normal Wnt signaling. We chose 293 cells because they have an intact Wnt network and are responsive to canonical ligands, including Wnt1 and Wnt3a [11], [12], [13]. Using quantitative real-time PCR (qPCR), we found that most of the Wnt receptors, including Fzd 1, 2, 3, 5, 6, 7, and 8, and LRP6, are expressed at detectable levels (Supplementary Figure S1A and Supplementary Table S1). Many of the Wnt ligands are also expressed, including Wnt3, Wnt5a, and Wnt5b (Supplementary Figure S1B and Supplementary Table S1). The expression of functional components of the network and the fact that there are no mutations or truncations in the genes encoding β-catenin or Adenomatous polyposis coli (APC), make these cells an attractive model to study normal Wnt signaling.

### Wnt3a induces survival, proliferation, and migration in HEK 293 cells

Most studies of Wnt signaling have used either Wnt3a-conditioned media or overexpression of Wnt3a to activate cells [13]. Both of these approaches activate canonical Wnt signaling, but the results are convoluted with the response of the cells to other growth factors present in the medium. To avoid this problem, we used purified recombinant human Wnt3a to activate signaling in serum-starved cells. To determine whether Wnt3a could activate the transcriptional program mediated by β-catenin/TCF, we used the well-characterized TOPglow and FOPglow reporter constructs [14]. TOPglow features the gene for firefly luciferase, under control of a TCF promoter; FOPglow features the same reporter gene, but downstream of a mutated TCF-binding site. As anticipated, luciferase activity was significantly increased in response to Wnt3a stimulation in TOPglow-transfected cells, but not in FOPglow-transfected cells (Supplementary Figure S2A). In addition, we observed a time-dependent increase in the overall levels of β-catenin by quantitative immunoblotting, which is a hallmark of canonical signaling, induced by Wnt3a [11], [13] (Supplementary Figure S2B). The increase in β-catenin was observed as early as five minutes after adding Wnt3a to serum-starved cells, suggesting that β-catenin levels are regulated post-transcriptionally (Supplementary Figure S2B). Using confocal imaging, we observed increased β-catenin at the membrane and in the cytosol upon Wnt3a stimulation, as previously reported [15], [16] (Supplementary Figure S2C).

The Wnt proteins are growth factors that have been shown to play critical roles in proliferation and migration [1], [17]. We found that Wnt3a promotes proliferation of serum-starved HEK 293 cells relative to unstimulated cells (Supplementary Figure S3A), consistent with previous studies [18], [19]. We also found that Wnt3a promotes cell survival, as serum-starvation-induced apoptosis was inhibited in Wnt3a-treated cells relative to untreated cells (Supplementary Figure S3B). Finally, using a Boyden chamber assay, we found that Wnt3a promotes cell migration (Supplementary Figure S3C). Together, these experiments establish HEK 293 cells as a suitable system to study Wnt3a-mediated signaling in the absence of the activating mutations found in most colorectal cancer cell lines.

### Monitoring the dynamics of gene expression reveals positive and negative feedback loops

**Figure 1:**
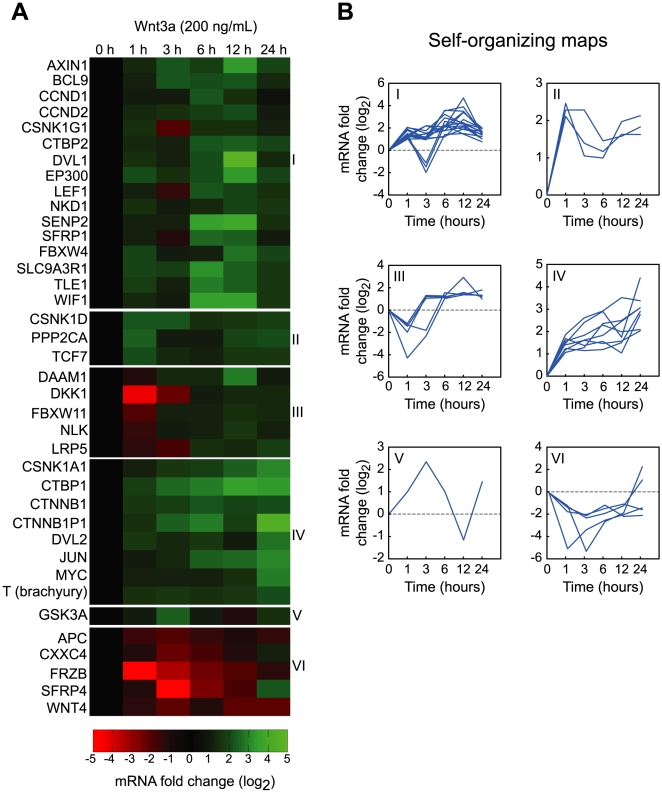
Monitoring the dynamics of Wnt3a-induced gene expression in HEK 293 cells reveals distinct time-dependent expression profiles. **A.** Heat map showing changes in the transcript levels of genes in the Wnt signaling network. HEK 293 cells were serum-starved for 24 hours and stimulated for different lengths of time with 200 ng/mL Wnt3a. Transcript levels were measured by qPCR and normalized to the average of five housekeeping genes. Changes are relative to serum-starved cells (0 h of stimulation). **B.** Genes with similar dynamic expression profiles were grouped together using self-organizing maps. Most canonical Wnt response genes fell into group IV, whereas negative regulators of Wnt signaling fell into groups III and VI.

To obtain a global view of gene regulation in the Wnt signaling network, we measured changes in the expression of 84 Wnt-related genes in Wnt3a-stimulated HEK 293 cells at six different time points over a 24-hour period using qPCR (Figure 1A and Supplementary Tables S2 and S3). Previous studies of the transcriptional response to Wnt ligands have focused on early (4 hour) and late (24 hour) treatments [15], [20]. Consistent with these studies, we observed Wnt3a-dependent increases in the transcript levels of canonical Wnt signaling genes at 3 and 6 hours, including *CTNNB1* (β-catenin) and *DVL1* (Dishevelled-1), as well as increases in canonical Wnt response genes at 24 hours, including *MYC* and *JUN*.

Simple inspection of our data, however, revealed a rich diversity in the time-dependent expression profiles (Figure 1A). To capture and classify the various response patterns, we used self-organizing maps. A self-organizing map is a type of artificial neural network that uses unsupervised learning to group similar vector data together. When we subjected our dynamic expression data to this analysis, we observed six distinct profiles (Figure 1B). Interestingly, most canonical Wnt response genes, including *MYC* and *JUN*, fell into group IV, which was characterized by a monotonic increase in mRNA levels over the entire 24-hour period, increasing rapidly in the first three hours and then leveling off. In contrast, the genes encoding negative regulators of Wnt signaling, such as secreted Fzd-related protein 4 (sFRP-4), Dickkopf-1 (DKK-1), Idax, and APC, fell into groups III and VI, which were both characterized by downregulation of mRNA levels in the first three hours, followed by subsequent increases. Thus the self-organizing maps highlighted an early positive feedback loop in which negative regulators of Wnt signaling are downregulated. Interestingly, both sFRP-4 and DKK-1 have been shown to play important roles in embryogenesis and oncogenesis [21]. Epigenetic silencing of *SFRP4* is frequently observed in colon cancer and usually occurs early in cancer progression [22], [23]. Additionally, it has been shown that restoring expression of *SFRP4* and *DKK-1* in colorectal cancer cells attenuates Wnt signaling, even in the presence of activating mutations in *CTNNB1* (β-catenin) and *APC* [23], [24].

**Figure 2:**
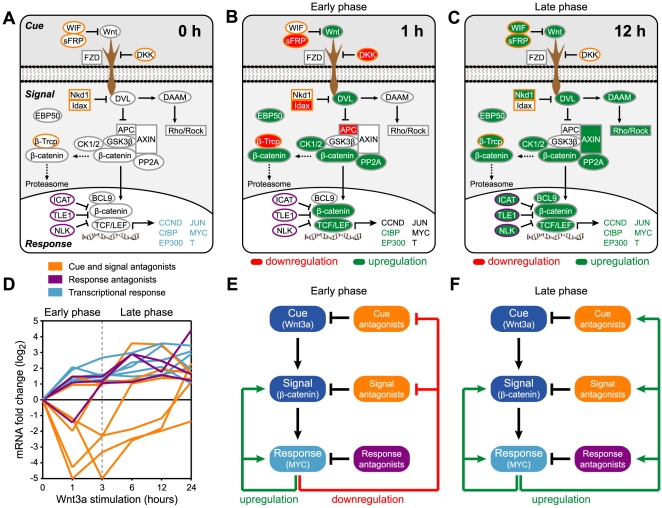
A knowledge-based view of the dynamics of gene expression reveals an early positive feedback loop, followed by a subsequent negative feedback loop. **A**. Simplified schematic of the canonical Wnt signaling network. Only proteins whose transcript levels changed by more than two-fold for at least one time point during the 24-hour time-course are shown. Cue and signal antagonists are outlined in orange; response antagonists are outlined in purple; transcriptional response genes are colored blue. **B**, **C**. Simplified schematics of the Wnt signaling network, highlighting proteins whose genes were upregulated (green) or downregulated (red) by more than two-fold after (B) 1 hour or (C) 12 hours of stimulation with 200 ng/mL Wnt3a. Schematics showing changes at all six time-points are provided in Figure S4. **D**. Plot of changes in the transcript levels of the proteins highlighted in panel (A). Cue and signal antagonists and shown in orange; response antagonists are shown in purple; transcriptional response genes are shown in blue. (E, F) The transcriptional response to Wnt3a stimulation is characterized by two distinct phases: an early phase (1–3 hours) and a late phase (3–24 hours). **E**. In the early phase, genes encoding cue and signal antagonists are downregulated, driving the upregulation of signal and response genes. **F**. In the late phase, genes encoding cue, signal, and response antagonists are upregulated, attenuating the expression of transcriptional response genes.

To obtain a biologically interpretable picture of gene regulation, we mapped significant (greater than two-fold) changes in mRNA levels onto a graphical depiction of the canonical Wnt signaling network (Figure 2, A–C). Proteins whose genes were upregulated are colored green, while those that were downregulated are colored red. For clarity, only three time-points are shown in Figure 2; all six time-points are provided in Supplementary Figure S4. Using the terminology of Lauffenburger and coworkers [25], the Wnt network can be viewed as comprising three informational layers: the ‘cue’ (extracellular ligands that activate the network), the ‘signal’ (intracellular proteins that transduce the message), and the ‘response’ (transcriptional program that effects a phenotype). Using this conceptual framework, we found that the Wnt3a-induced transcriptional program is characterized by two distinct phases: an early phase (0–3 hours) and a late phase (3–24 hours). In the early phase, antagonists of the cue and signal are downregulated (Figure 2, D–E). This is accompanied by a sharp increase in the expression of signal and response genes. In the late phase, antagonists of the cue, the signal, and the response are upregulated and the transcription of response genes levels off or even diminishes (Figure 2, D and F). Thus the early phase of Wnt signaling is characterized by a transient positive feedback loop that promotes the expression of signal and response genes, whereas the late phase is characterized by a negative feedback loop that attenuates signaling.

Using quantitative Western blotting, we also examined time-dependent changes in the abundance of eight Wnt signaling proteins upon stimulation of HEK 293 cells with Wnt3a. Consistent with our transcriptional data and with previous studies [16], we observed a Wnt3a-induced increase in the levels of β-catenin, Naked1, Myc, CtBP1, CK1 and Dvl3 (Supplementary Figure S5). As expected, changes in protein levels lagged slightly behind the observed changes in transcript levels. Consistent with our mRNA data, we also observed decreases in the abundances of negative regulators of Wnt signaling, such as sFRP4 and DKK-1, during the early phase of Wnt3a stimulation, and increases in their abundances during the later phase (Supplementary Figure S5). Mechanistically, sFRPs function as soluble antagonists of Wnt signaling by binding directly to Wnt proteins and preventing their interactions with Frizzled receptors[26], [27]. Similarly, DKK-1 is a secreted antagonist of Wnt signaling that binds directly to and inhibits the Wnt co-receptor LRP6 [28]. The downregulation of these negative regulators, along with the upregulation of positive regulators such as β-catenin, Myc, and CtBP1, combined to augment Wnt signaling in the first three hours and hence provide positive feedback. These same negative regulators are downregulated at later time points, thereby attenuating signaling.

### c-Myc and CtBP1 are overexpressed in colorectal adenocarcinomas

**Figure 3:**
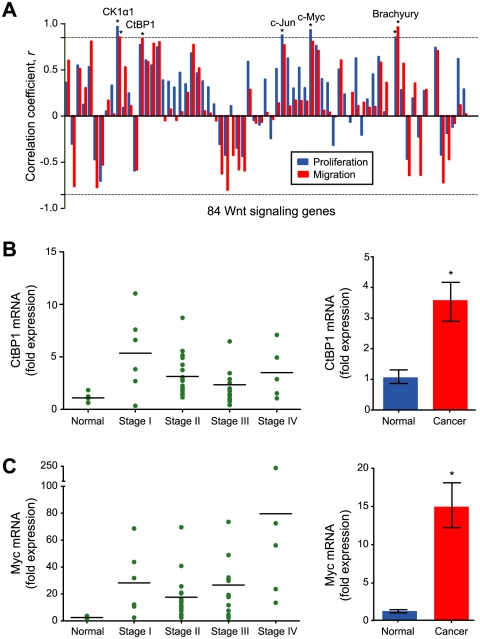
Correlations between dynamic expression profiles and cellular phenotypes identify response genes that are significantly overexpressed in colorectal carcinomas. **A**. Correlations were calculated between time-dependent expression profiles and either proliferation (blue) or migration (red). The dotted line indicates a *P*-value of 0.05 (* *P*<0.05). **B**, **C**. Transcript levels of CtBP1 and c-Myc in human colorectal carcinomas. Left: the relative levels of CtBP1 or c-Myc mRNA in normal colon tissue and in colorectal cancers of different stages. Horizontal bars indicate the mean of each group. Right: the relative levels of CtBP1 and c-Myc mRNA were significantly higher in colon cancer tissue relative to normal mucosa (* *P*<0.05; Kruskal-Wallis test). Error bars indicate the standard deviation. Data for Casein kinase 1α1, c-Jun, and Brachyury are provided in Figure S6.

Having identified these novel phases of transcriptional regulation, we asked if our dynamic expression profiles could be used to identify genes that drive cellular proliferation and migration. We argued that genes with time-dependent profiles that most closely correlated with our quantitative measurements of proliferation and migration (Supplementary Figure S3, A and C) are most likely to act in a causal fashion. As correlation is not same as causation, however, further experiments are required to establish causation in each case. Using linear regression, we identified five genes with profiles that correlated significantly (*P*<0.05) with either proliferation, migration, or both: *MYC* (c-Myc), *JUN* (c-Jun), *CSNK1A1* (Casein kinase 1α1), *CTBP1* (C-terminal binding protein 1), and *T* (Brachyury) (Figure 3A). All five genes are transcriptional response genes and are found in self-organizing map IV (Figure 1B). c-Myc and c-Jun are transcription factors that are well known to drive cycle progression and are prototypical targets of canonical Wnt signaling [9]. In addition, several isoforms of casein kinase 1 (CK1) have been shown to positively regulate Wnt signaling [29]. The roles of CtBP1 and Brachyury, however, are less well defined.

**Figure 4:**
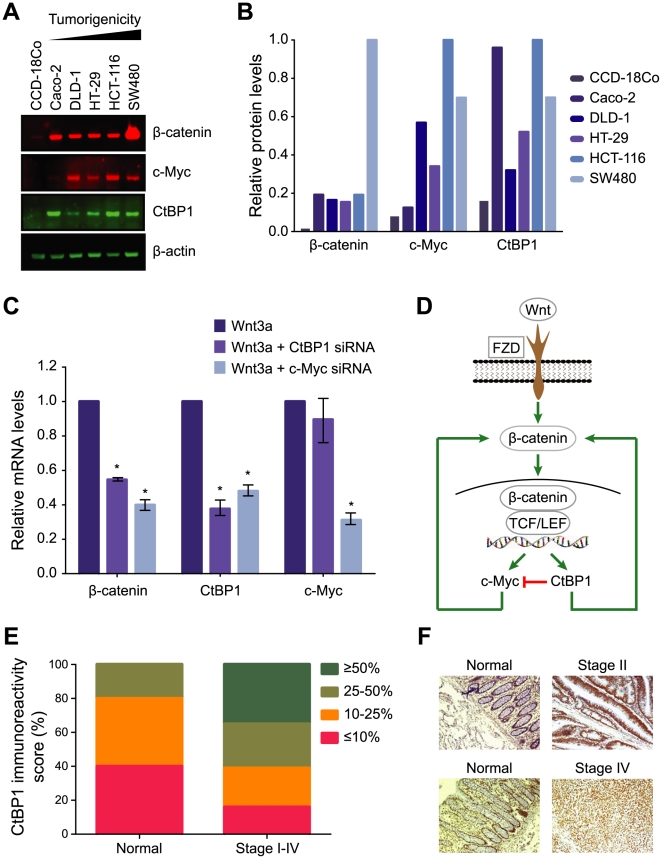
CtBP1 and c-Myc upregulate β-catenin and are markers of tumor progression in colorectal carcinomas. **A**. Western blots reveal progressively higher levels of β-catenin, c-Myc, and CtBP1 in colon cancer cell lines of increasing tumorigenicity (Caco-2<DLD-1<HT-29<HCT116<SW480), relative to the normal colon cell line, CCD-18Co. **B**. Quantification of the Western blot data in panel A. All values were normalized to β-actin levels. **C**. Roles of CtBP1 and c-Myc in regulating gene expression. HEK 293 cells were transiently transfected with control siRNA or siRNAs targeting either CtBP1 or c-Myc. After 48 hours, the cells were serum-starved for 24 hours and stimulated with 200 ng/mL Wnt3a for 12 hours. Transcript levels were measured by qPCR. Knocking down either CtBP1 or c-Myc resulted in decreased levels of β-catenin. Data are the mean of two independent experiments and error bars indicate the standard error of the mean (* *P*<0.05; one-way ANOVA). **D**. Pathway diagram illustrating the roles of CtBP1 and c-Myc in Wnt3a-mediated activation of canonical Wnt signaling. The levels of both CtBP1 and c-Myc are upregulated in response to Wnt3a stimulation. In the absence of Wnt3a, CtBP1 acts a transcriptional repressor and inhibits the expression of *MYC*. In the presence of Wnt3a, both CtBP1 and c-Myc drive Wnt signaling by upregulating β-catenin. **E**. Distribution of CtBP1 immunoreactivity scores in normal mucosa and in colorectal adenocarcinomas determined using tissue microarrays (*n* = 75 cases; 150 tumor cores). The immunoreactivity of CtBP1 was classified using a four-grade scale: 0, fewer than 10% positively stained cells; 1, between 10% and 25%; 2, between 25% and 50%; and 3, greater than 50%. **F**. Representative images from the tissue microarrays, showing patient-matched normal and cancerous tissue from stage II and stage IV colorectal carcinomas (10× magnification).

Since aberrant Wnt signaling is strongly tied to colorectal cancer, we measured the transcript levels of all five genes in 48 tissue samples obtained from patients with histopathologically confirmed colon cancer (stage I, *n* = 6; stage II, *n* = 18; stage III, *n* = 14; stage IV, *n* = 5), as well as in normal colon samples (*n* = 5). We found that *MYC*, *JUN*, and *CTBP1* were significantly overexpressed in all four stages of colorectal cancer compared with normal tissue (*P*<0.05) (Figure 3, B and C, and Supplementary Figure S6A). In contrast, *CSNK1A1* and *T* were not significantly overexpressed (Supplementary Figure S6, B and C). Consistent with these observations, we observed substantially higher levels of c-Myc, CtBP1, and β-catenin in colon cancer cell lines with constitutively active Wnt signaling than in CCD-18Co, a normal colon fibroblast cell line [30] (Figure 4, A and B). In addition, the levels of c-Myc and CtBP1 roughly tracked with the tumorigenicity of the cell lines.

CtBP1 can act as both a transcriptional co-repressor and a transcriptional activator [31], [32], [33], [34], [35]. It has been shown to promote epithelial-mesenchymal transition (EMT) and to function as an antagonist of apoptosis [36]. To further investigate the roles of CtBP1 and c-Myc in driving Wnt signaling, we knocked down their transcript levels by RNAi and measured the mRNA levels of β-catenin, CtBP1, and c-Myc after 12 hours of stimulation with Wnt3a. It has previously been suggested that c-Myc can promote Wnt signaling by upregulating β-catenin. Consistent with this notion, we found that β-catenin mRNA levels were significantly decreased when c-Myc was knocked down (*P*<0.05) (Figure 4C). Interestingly, the same result was observed when we knocked down CtBP1, indicating that it also promotes Wnt signaling through a positive feedback loop (Figures 4C and 4D). We also found that knocking down the levels of c-Myc led to a significant reduction in the levels of CtBP1 mRNA (*P*<0.05). As the Wnt3a-dependent upregulation of *CTBP1* precedes the upregulation of *MYC* (Figure 2B and Supplementary Table S3), we conclude that c-Myc promotes the expression of *CTBP1* indirectly by upregulating β-catenin (Figure 4D). Knocking down the levels of CtBP1 did not significantly affect c-Myc transcript levels. Previous studies have shown, however, that in the absence of Wnt stimulation, CtBP1 interacts with TCF-4 to represses the expression of *MYC* [37]. Thus, we submit that knocking down CtBP1 has counteracting effects: it leads to decreased Wnt-independent repression of *MYC* expression as well as decreased Wnt-dependent upregulation of β-catenin. Overall, we conclude that c-Myc and CtBP1 are largely independent response genes that promote Wnt signaling by upregulating β-catenin.

To further investigate the role of CtBP1 in colorectal cancer, we used tissue microarrays to assess the levels of the CtBP1 protein in normal mucosa, as well as in adenocarcinoma tissue obtained from 75 patients with colorectal cancer (Figure 4, E and F). As representative examples, Figure 4F shows CtBP1 staining in stage II and stage IV cancers, along with normal tissue obtained from the same patient. Overall, we evaluated 150 tumor cores for CtBP1 staining and found that more than half of them exhibited higher immunoreactivity scores in the tumor samples than in the patient-matched normal tissue (Figure 4E). These data are consistent with our *in vitro* observation that CtBP1 drives Wnt signaling and identifies CtBP1 as a potential marker for colon cancer progression. Our data are also consistent with a recent study in zebrafish embryos that showed that loss of APC and accumulation of CtBP1 represent early steps in cancer progression (formation of adenomas), whereas nuclear localization of β-catenin represents a later stage (carcinogenesis) [38]. Our time-dependent expression profile highlights upregulation of CtBP1 as an early event following activation of Wnt signaling and our subsequent analysis of human colorectal tumors showed that CtBP1 is overexpressed in early, as well as late-stage, cancers.

In summary, our system-wide and quantitative characterization of transcriptional regulation uncovered previously unrecognized positive and negative feedback loops that reveal how this network is initially activated and subsequently attenuated. Further, these time-dependent analyses highlighted CtBP1, a nuclear protein that falls outside the canonical Wnt pathway and is overexpressed in all four stages of colorectal adenocarcinoma. Identifying noncanonical components of the Wnt network that drive tumorigenesis may provide novel strategies for developing effective therapeutics in a network that, to date, has largely eluded pharmaceutical discovery.

## Materials and Methods

### Ethics Statement

N/A. An ethics statement is not required for this work. An informed consent from participants involved is also not applicable for this work.

### Cell lines and reagents

HEK Flp-In-293 cells were obtained from Invitrogen (Carlsbad, CA) and maintained in Dulbecco's Modified Eagle Medium (DMEM) supplemented with 10% (v/v) fetal bovine serum (FBS), 2 mM glutamine, 100 IU/mL penicillin, and 100 µg/mL streptomycin. Colon cancer cell lines (CCD-18co, Caco-2, DLD-1, HT-29, HCT-116, and SW480) were obtained from American Type Culture Collection (ATCC, Rockville, MD) and maintained in DMEM supplemented with 10% FBS. Signal Silence c-Myc siRNA was from Cell Signaling Technology (Danvers, MA) and CtBP1 siRNA was from Santa Cruz Biotechnology (Santa Cruz, CA). They were both introduced into cells using Lipofectamine 2000 (Invitrogen) according to the manufacturer's instructions. Recombinant human Wnt3a was from R&D Systems (Minneapolis, MN). Primary antibodies were obtained from the following sources: rabbit anti-β-catenin (Cell Signaling Technology; cat. #9562); rabbit anti-c-Myc (Cell Signaling Technology; cat. #9402); rabbit anti-Naked1 (Cell Signaling Technology; cat. #2262); rabbit anti-CK1 (Cell Signaling Technology; cat. #2655); rabbit anti-Dvl3 (Cell Signaling Technology; cat. #3218); rabbit anti-GSK3β (Cell Signaling Technology; cat. #9315); rabbit anti-sFrp4 (Upstate Biotechnology Inc., Lake Placid, NY); cat #09-129); mouse anti-Dkk-1(Millipore; cat #MAB10132); mouse anti-β-actin (Sigma-Aldrich, Inc., St. Louis, MO; cat. #A1978); and mouse anti-CtBP1 (Santa Cruz Biotechnology, Inc., Santa Cruz, CA; cat. #SC-17805).

### Protein isolation and quantitative Western blotting

HEK 293 cells were serum-starved for 24 h and stimulated with Wnt3a (200 ng/mL) for the appropriate length of time. Cells were rinsed in Phosphate Buffered Saline (PBS) and lysed in Lysis Buffer (20 mM Tris-HCl, 150 mM NaCl, 1% Triton X-100 (v/v), 2 mM EDTA, pH 7.8 supplemented with 1 mM sodium orthovanadate, 1 mM phenylmethylsulfonyl fluoride (PMSF), 10 µg/mL aprotinin, and 10 µg/mL leupeptin). Protein concentrations were determined using the BCA protein assay (Pierce, Rockford, IL) and immunoblotting experiments were performed using standard procedures. For quantitative immunoblots, primary antibodies were detected with IRDye 680-labeled goat-anti-rabbit IgG or IRDye 800-labeled goat-anti-mouse IgG (LI-COR Biosciences, Lincoln, NE) at 1∶10,000 dilution. Bands were visualized and quantified using an Odyssey Infrared Imaging System (LI-COR Biosciences).

### RNA extraction and quantitative real-time PCR

HEK 293 cells were serum-starved for 24 h and stimulated with Wnt3a (200 ng/mL) for the appropriate length of time. Total cellular RNA was isolated using an RNeasy Mini Kit (QIAGEN, Santa Clara, CA). mRNA levels for the 84 Wnt-related genes were determined using the RT^2^ profiler™ qPCR array (SA Biosciences Corporation, Frederick, MD). Briefly, 1 µg of total RNA was reverse transcribed into first strand cDNA using an RT^2^ First Strand Kit (SA Biosciences). The resulting cDNA was subjected to qPCR using human gene-specific primers for 96 different genes, including the 84 Wnt-related genes and five housekeeping genes (*B2M*, *HPRT1*, *RPL13A*, *GAPDH*, and *ACTB*). The qPCR reaction was performed with an initial denaturation step of 10 min at 95°C, followed by 15 s at 95°C and 60 s at 60°C for 40 cycles using an Mx3000P™ QPCR system (Stratagene, La Jolla, CA).

The mRNA levels of each gene were normalized relative to the mean levels of the five housekeeping genes and compared with the data obtained from unstimulated, serum-starved cells using the 2^−ΔΔ*Ct*^ method. According to this method, the normalized level of a mRNA, *X*, is determined using equation 1:
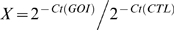
(1)where *Ct* is the threshold cycle (the number of the cycle at which an increase in reporter fluorescence above a baseline signal is detected), GOI refers to the gene of interest, and CTL refers to a control housekeeping gene. This method assumes that *Ct* is inversely proportional to the initial concentration of mRNA and that the amount of product doubles with every cycle. To determine the fold-change in gene expression induced by stimulation with Wnt3a, the normalized expression of each gene in the Wnt3a-stimulated sample was divided by the normalized expression of the same gene in the unstimulated sample. The qPCR data presented are mean of three biological replicates. Only gene whose mean transcript levels changed by more than two-fold for at least one time point during the 24-hour time-course were considered significant. A self-organizing map was generated with the Spotfire program to cluster genes with self-similar expression profiles.

### TOPglow/FOPglow reporter assay

For dual luciferase reporter assays, HEK 293 cells were co-transfected with either TOPglow or FOPglow (Millipore, Billerica, MA) and the pRL-TK control vector (Promega, Madison, WI). After 48 h, cells were stimulated with 200 ng/mL Wnt3a for 24 h and luciferase activity was measured using a dual luciferase reporter kit (Promega).

### Cell proliferation assay

Cell proliferation was measured using a BrdU proliferation assay kit (Calbiochem, San Diego, CA). Briefly, cells were seeded onto 96-well plates, serum-starved for 24 h, and incubated with BrdU for 24 h. During the 24-h incubation, 200 ng/mL Wnt3a was added at different times. At the end of the time course, cells were fixed and analyzed for BrdU incorporation. Measurements were performed in triplicate and normalized to untreated cells.

### Cell migration assay

Cell migration was assessed using a QCM™ chemotaxis 96-well cell migration assay kit (Chemicon, Temecula, CA). Briefly, HEK 293 cells were suspended in DMEM and plated in the top chamber. DMEM containing 200 ng/mL Wnt3a was added to the bottom chamber for different lengths of time. Migratory cells in the bottom chamber were dissociated from the membrane, lysed, and quantified by adding CyQuant GR dye. Measurements were performed in triplicate and normalized to untreated cells.

### Confocal microscopy

HEK 293 cells were cultured on Lab-Tek II chamber glass slides (Nalge Nunc, Naperville, IL), serum-starved for 24 h, and treated with 200 ng/mL Wnt3a for different lengths of time. Cells were then fixed in 4% paraformaldehyde for 40 min at room temperature, washed in PBS, permeabilized with 0.15% Triton X-100, and blocked for 30 min with PBS containing 3% bovine serum albumin (BSA). Cells were immunostained with a rabbit anti-β-catenin antibody, following by an Alexa Fluor 488-labeled goat-anti-rabbit antibody (Molecular Probes, Eugene, OR). The nuclei were counterstained with Hoescht 33342 (Sigma-Aldrich, St. Louis, MO). Fluorescent micrographs were obtained using a Zeiss LSM510 META confocal microscope (Carl Zeiss, Jena, Germany). Individual channels were overlaid using Image J software, and localization was determined using the Image J RG2B co-localization plug-in.

### Apoptosis assays

HEK 293 cells were serum-starved for 24 h, stimulated with 200 ng/mL Wnt3a, harvested, fixed in absolute ethanol, and treated with RNaseA at 37°C overnight. The cells were then incubated in 5 mg/mL of propidium iodide for 15 min at room temperature and cell cycle analysis was performed using a BD LSRII flow cytometer (BD Biosciences, San Jose, CA). Measurements were performed in triplicate and statistical significance was calculated by one-way ANOVA.

### TissueScan oncology panel arrays

A collection of 48 cDNA samples derived from tumor biopsies was obtained from OriGene Technologies Inc. (Rockville, MD). The samples represented all four stages of colon carcinoma, as well as normal tissue. Gene expression was assessed by qPCR as described above. The cancer data were normalized relative to the data collected from the normal tissue samples and analyzed using the Kruskal-Wallis test at a significance level of 0.05.

### Tissue microarrays and immunohistochemistry

Two tissue microarrays featuring colon cancer with matching normal mucosa were purchased from Pantomics, Inc. (Richmond, CA). The first array comprised 16 cases of colon cancer, each in duplicate, with corresponding normal tissue from the same patient as a control. The other array comprised tissue, in duplicate, from 75 individuals, representing both normal colon and different grades and stages of colon cancer. Immunohistochemistry was performed as previously described [39]. For negative controls, primary antibody was omitted. The percentage of positive cells was assessed visually in each core and the intensity of CtBP1 staining was classified using a four-grade scale: 0 indicated that fewer than 10% of the cells were positive; 1, between 10% and 25%; 2, between 25% and 50%; and 3, greater than 50%.

## Supporting Information

Figure S1 Relative mRNA levels of (A) Wnt receptors and (B) Wnt ligands in HEK 293 cells. Total RNA was isolated from serum-starved HEK 293 cells and mRNA levels were determined by qPCR using gene-specific primers. All data are the mean of two independent experiments and error bars indicate the standard error of the mean (SEM).
(0.65 MB EPS)

Figure S2 Wnt3a stimulation activates canonical Wnt signaling in HEK 293 cells. (A) Wnt3a stimulation activates transcription from the TCF promoter. HEK 293 cells were transiently transfected with pRL-TK reporters and either TOPglow or FOPglow. After stimulating the cells with 200 ng/mL Wnt3a, a dual-luciferase assay was performed. Wnt3a induced transcription in the TOPglow-transfected cells, but not in the FOPglow-transfected cells (negative control). Data are the mean of two independent experiments and error bars indicate the standard error of the mean (SEM). (B) Western blot showing that Wnt3a induces increased levels of β-catenin. Serum-starved HEK 293 cells were stimulated with 200 ng/mL Wnt3a and whole cell lysates were collected at the indicated times. Western blots were probed with antibodies directed at β-catenin and β-actin (loading control). The bar graph provides relative levels of β-catenin, normalized to β-actin levels. The error bars indicate SEM of two biological repeats. (C) Localization of β-catenin in HEK 293 cells. Serum-starved HEK 293 cells were stimulated with 200 ng/mL Wnt3a for the indicated times and fixed in 4% paraformaldehyde. Cells were stained with Hoechst dye (DNA; blue) and with an anti-β-catenin antibody (red).
(1.91 MB EPS)

Figure S3 Wnt3a stimulation promotes proliferation, survival, and migration in HEK 293 cells. (A) Wnt3a stimulation induced proliferation of HEK 293 cells over a 24-hour period. HEK 293 cells were serum-starved for 24 hours and incubated with BrdU for 24 hours. Wnt3a was added to the cells at the indicated times before the end of the 24-hour BrdU treatment. Data are the mean of three independent experiments and error bars indicate the standard error of the mean (SEM). (B) Wnt3a inhibits serum-starvation-induced apoptosis. HEK 293 cells were serum-starved for 24 hours and treated with Wnt3a for the indicated times. Cell were fixed and stained with propidium iodide. The percentage of apoptotic cells was measured by flow cytometry. Data are the mean of two independent experiments and error bars indicate the SEM. (C) Wnt3a induces migration of HEK 293 cells. HEK 293 cells were serum-starved for 24 hours and plated in the upper chamber of a QCM™ chemotaxis 96-well cell migration plate while Wnt3a was added to the lower chamber. Migration of cells from the upper to the lower chamber was measured using CyQuant GR dye. Data are the mean of three independent experiments and error bars indicate the SEM.
(0.96 MB EPS)

Figure S4 A knowledge-based view of the dynamics of gene expression in the Wnt signaling network. (A) Simplified schematic of the canonical Wnt signaling network. Only proteins whose transcript levels changed by more than two-fold are shown. Cue and signal antagonists are outlined in orange; response antagonists are outlined in purple; transcriptional response genes are colored blue. (B–F) Simplified schematics of the Wnt network, highlighting proteins whose genes were upregulated (green) or downregulated (red) by more than two-fold after stimulation with 200 ng/mL Wnt3a for different lengths of time.
(3.72 MB EPS)

Figure S5 Changes in protein abundance upon stimulation of HEK 293 cells with Wnt3a. (A) Simplified schematic of the canonical Wnt signaling network. Proteins whose levels were monitored by quantitative Western blotting are highlighted in orange. (B) Dynamic profiles of eight Wnt signaling proteins following stimulation with Wnt3a. Whole cell lysates were analyzed by Western blotting and protein levels were normalized to β-actin levels. Relative protein levels were calculated for each protein by first subtracting the level observed in the untreated sample and then dividing by the maximum oberved value. All data are the mean of at least two independent experiments and error bars indicate the standard error of the mean (SEM).
(2.22 MB EPS)

Figure S6 Transcript levels of c-Jun, CK1α1, and Brachyury in human colorectal carcinomas. Left: the relative levels of (A) c-Jun, (B) CK1α1, and (C) Brachyury mRNA in normal colon tissue and in colorectal cancers of different stages were measured by qPCR. Horizontal bars indicate the mean of each group. Right: the relative levels of c-Jun mRNA were significantly higher in colon cancer tissue relative to normal mucosa (* P<0.05; Kruskal-Wallis test). The relative leves of CK1α1 and Brachyury mRNA were not significantly higher. Error bars indicate the standard deviation.
(1.75 MB EPS)

Table S1 Expression levels of Wnt receptors and Wnt ligands in HEK 293 cells. The Ct values are the means of at least two biological replicates.
(0.04 MB DOC)

Table S2 Wnt signaling genes assessed by quantitative real-time PCR.
(0.09 MB DOC)

Table S3 Relative changes in the expression levels of Wnt signaling genes in response to stimulation with Wnt3a.
(0.12 MB DOC)
