# Supplementary material for: A System-Wide Investigation of the Dynamics of Wnt Signaling Reveals Novel Phases of Transcriptional Regulation
Source: PLoS One. 2010 Apr 7;5(4):e10024. doi: 10.1371/journal.pone.0010024 (PMC2850918; doi:10.1371/journal.pone.0010024)
Supplement: Table S1 — Expression levels of Wnt receptors and Wnt ligands in HEK 293 cells. The Ct values are the means of at least two biological replicates. (0.04 MB DOC) [file pone.0010024.s007.doc]

**Supplementary Table S1.** Expression levels of Wnt receptors and Wnt ligands in HEK 293 cells. The *Ct* values are the means of at least two biological replicates.

| **Gene** | **Mean *Ct* Value** |
| --- | --- |
| **Wnt Receptors** | |
| FZD1 | 23.09 |
| FZD2 | 24.16 |
| FZD3 | 25.38 |
| FZD4 | 27.74 |
| FZD5 | 23.51 |
| FZD6 | 23.49 |
| FZD7 | 23.60 |
| FZD8 | 21.63 |
| LRP5 | 28.69 |
| LRP6 | 23.48 |
| **Wnt Ligands** | |
| WNT1 | 32.47 |
| WNT2 | 28.70 |
| WNT2B | 27.49 |
| WNT3 | 22.46 |
| WNT3A | 30.08 |
| WNT4 | 26.12 |
| WNT5A | 23.04 |
| WNT5B | 25.26 |
| WNT6 | No Ct |
| WNT7A | 32.14 |
| WNT7B | 27.02 |
| WNT8A | 28.94 |
| WNT9A | 25.90 |
| WNT10A | 30.35 |
| WNT11 | 28.99 |
| WNT16 | 26.88 |
