# Supplementary material for: A System-Wide Investigation of the Dynamics of Wnt Signaling Reveals Novel Phases of Transcriptional Regulation
Source: PLoS One. 2010 Apr 7;5(4):e10024. doi: 10.1371/journal.pone.0010024 (PMC2850918; doi:10.1371/journal.pone.0010024)
Supplement: Table S2 — Wnt signaling genes assessed by quantitative real-time PCR. (0.09 MB DOC) [file pone.0010024.s008.doc]

**Supplementary Table S2. Wnt signaling genes assessed by quantitative real-time PCR.**

| **Gene Symbol** | **Protein** | **NCBI Reference Sequence** |
| --- | --- | --- |
| *AES* | Amino-terminal enhancer of split | NM_001130 |
| *APC* | Adenomatous polyposis coli | NM_000038 |
| *AXIN1* | Axin 1 | NM_003502 |
| *BCL9* | B-cell CLL/lymphoma 9 | NM_004326 |
| *BTRC* | Beta-transducin repeat containing | NM_033637 |
| *FZD5* | Frizzled homolog 5 (Drosophila) | NM_003468 |
| *CCND1* | Cyclin D1 | NM_053056 |
| *CCND2* | Cyclin D2 | NM_001759 |
| *CCND3* | Cyclin D3 | NM_001760 |
| *CSNK1A1* | Casein kinase 1, alpha 1 | NM_001892 |
| *CSNK1D* | Casein kinase 1, delta | NM_001893 |
| *CSNK1G1* | Casein kinase 1, gamma 1 | NM_022048 |
| *CSNK2A1* | Casein kinase 2, alpha 1 polypeptide | NM_001895 |
| *CTBP1* | C-terminal binding protein 1 | NM_001328 |
| *CTBP2* | C-terminal binding protein 2 | NM_022802 |
| *CTNNB1* | Catenin (cadherin-associated protein), beta 1, 88 kDa | NM_001904 |
| *CTNNBIP1* | Catenin, beta interacting protein 1 | NM_020248 |
| *CXXC4* | CXXC finger 4 | NM_025212 |
| *DAAM1* | Dishevelled associated activator of morphogenesis 1 | NM_014992 |
| *DIXDC1* | DIX domain containing 1 | NM_033425 |
| *DKK1* | Dickkopf homolog 1 (Xenopus laevis) | NM_012242 |
| *DVL1* | Dishevelled, dsh homolog 1 (Drosophila) | NM_004421 |
| *DVL2* | Dishevelled, dsh homolog 2 (Drosophila) | NM_004422 |
| *EP300* | E1A binding protein p300 | NM_001429 |
| *FBXW11* | F-box and WD repeat domain containing 11 | NM_012300 |
| *FBXW2* | F-box and WD repeat domain containing 2 | NM_012164 |
| *FGF4* | Fibroblast growth factor 4 | NM_002007 |
| *FOSL1* | FOS-like antigen 1 | NM_005438 |
| *FOXN1* | Forkhead box N1 | NM_003593 |
| *FRAT1* | Frequently rearranged in advanced T-cell lymphomas | NM_005479 |
| *FRZB* | Frizzled-related protein | NM_001463 |
| *FSHB* | Follicle stimulating hormone, beta polypeptide | NM_000510 |
| *FZD1* | Frizzled homolog 1 (Drosophila) | NM_003505 |
| *FZD2* | Frizzled homolog 2 (Drosophila) | NM_001466 |
| *FZD3* | Frizzled homolog 3 (Drosophila) | NM_017412 |
| *FZD4* | Frizzled homolog 4 (Drosophila) | NM_012193 |
| *FZD6* | Frizzled homolog 6 (Drosophila) | NM_003506 |
| *FZD7* | Frizzled homolog 7 (Drosophila) | NM_003507 |
| *FZD8* | Frizzled homolog 8 (Drosophila) | NM_031866 |
| *GSK3A* | Glycogen synthase kinase 3 alpha | NM_019884 |
| *GSK3B* | Glycogen synthase kinase 3 beta | NM_002093 |
| *JUN* | Jun oncogene | NM_002228 |
| *KREMEN1* | Kringle containing transmembrane protein 1 | NM_001039570 |
| *LEF1* | Lymphoid enhancer-binding factor 1 | NM_016269 |
| *LRP5* | Low density lipoprotein receptor-related protein 5 | NM_002335 |
| *LRP6* | Low density lipoprotein receptor-related protein 6 | NM_002336 |
| *MYC* | V-myc myelocytomatosis viral oncogene homolog (avian) | NM_002467 |
| *NKD1* | Naked cuticle homolog 1 (Drosophila) | NM_033119 |
| *NLK* | Nemo-like kinase | NM_016231 |
| *PITX2* | Paired-like homeodomain 2 | NM_000325 |
| *PORCN* | Porcupine homolog (Drosophila) | NM_022825 |
| *PPP2CA* | Protein phosphatase 2 (formerly 2A), catalytic subunit, alpha isoform | NM_002715 |
| *PPP2R1A* | Protein phosphatase 2 (formerly 2A), regulatory subunit A, alpha isoform | NM_014225 |
| *PYGO1* | Pygopus homolog 1 (Drosophila) | NM_015617 |
| *RHOU* | Ras homolog gene family, member U | NM_021205 |
| *SENP2* | SUMO1/sentrin/SMT3 specific peptidase 2 | NM_021627 |
| *SFRP1* | Secreted frizzled-related protein 1 | NM_003012 |
| *SFRP4* | Secreted frizzled-related protein 4 | NM_003014 |
| *FBXW4* | F-box and WD repeat domain containing 4 | NM_022039 |
| *SLC9A3R1* | Solute carrier family 9 (sodium/hydrogen exchanger), member 3 regulator 1 | NM_004252 |
| *SOX17* | SRY (sex determining region Y)-box 17 | NM_022454 |
| *T* | T, brachyury homolog (mouse) | NM_003181 |
| *TCF7* | Transcription factor 7 (T-cell specific, HMG-box) | NM_003202 |
| *TCF7L1* | Transcription factor 7-like 1 (T-cell specific, HMG-box) | NM_031283 |
| *TLE1* | Transducin-like enhancer of split 1 (Esp1 homolog, Drosophila) | NM_005077 |
| *TLE2* | Transducin-like enhancer of split 2 (Esp1 homolog, Drosophila) | NM_003260 |
| *WIF1* | WNT inhibitory factor 1 | NM_007191 |
| *WISP1* | WNT1 inducible signaling pathway protein 1 | NM_003882 |
| *WNT1* | Wingless-type MMTV integration site family, member 1 | NM_005430 |
| *WNT10A* | Wingless-type MMTV integration site family, member 10A | NM_025216 |
| *WNT11* | Wingless-type MMTV integration site family, member 11 | NM_004626 |
| *WNT16* | Wingless-type MMTV integration site family, member 16 | NM_057168 |
| *WNT2* | Wingless-type MMTV integration site family member 2 | NM_003391 |
| *WNT2B* | Wingless-type MMTV integration site family, member 2B | NM_004185 |
| *WNT3* | Wingless-type MMTV integration site family, member 3 | NM_030753 |
| *WNT3A* | Wingless-type MMTV integration site family, member 3A | NM_033131 |
| *WNT4* | Wingless-type MMTV integration site family, member 4 | NM_030761 |
| *WNT5A* | Wingless-type MMTV integration site family, member 5A | NM_003392 |
| *WNT5B* | Wingless-type MMTV integration site family, member 5B | NM_032642 |
| *WNT6* | Wingless-type MMTV integration site family, member 6 | NM_006522 |
| *WNT7A* | Wingless-type MMTV integration site family, member 7A | NM_004625 |
| *WNT7B* | Wingless-type MMTV integration site family, member 7B | NM_058238 |
| *WNT8A* | Wingless-type MMTV integration site family, member 8A | NM_058244 |
| *WNT9A* | Wingless-type MMTV integration site family, member 9A | NM_003395 |
| *B2M* | Beta-2-microglobulin | NM_004048 |
| *HPRT1* | Hypoxanthine phosphoribosyltransferase 1 (Lesch-Nyhan syndrome) | NM_000194 |
| *RPL13A* | Ribosomal protein L13a | NM_012423 |
| *GAPDH* | Glyceraldehyde-3-phosphate dehydrogenase | NM_002046 |
| *ACTB* | Actin, beta | NM_001101 |
