# Supplementary material for: A System-Wide Investigation of the Dynamics of Wnt Signaling Reveals Novel Phases of Transcriptional Regulation
Source: PLoS One. 2010 Apr 7;5(4):e10024. doi: 10.1371/journal.pone.0010024 (PMC2850918; doi:10.1371/journal.pone.0010024)
Supplement: Table S3 — Relative changes in the expression levels of Wnt signaling genes in response to stimulation with Wnt3a. (0.12 MB DOC) [file pone.0010024.s009.doc]

**Supplementary Table S3. Relative changes^a^ in the expression levels of Wnt signaling genes in response to stimulation with Wnt3a.**

| **Gene** | **Stimulation Time (hours)**  **1 3 6 12 24** | | | | |
| --- | --- | --- | --- | --- | --- |
| *AES* | 1.1487 | 1.2363 | 1.458 | 1.0238 | 1.434 |
| *APC* | -1.7171 | -1.9643 | -1.4201 | -1.0253 | -1.5263 |
| *AXIN1* | 1.366 | 2.1977 | 1.7581 | 3.3964 | 2 |
| *BCL9* | 1.1974 | 2.2038 | 2.08493 | 2.1886 | 1.3851 |
| *BTRC* | 1.28342 | 1.181 | 1.0867 | 1.1329 | 1.3013 |
| *FZD5* | -1.5911 | -1.0644 | -1.3755 | -1.1019 | -1.5911 |
| *CCND1* | 1 | 1.042466 | 2.250117 | 1.453973 | 0.76313 |
| *CCND2* | 1.257 | 1.4702 | 1.7581 | 2.0907 | 1.0281 |
| *CCND3* | -1.0792 | 1.0396 | 1.2605 | 1.1843 | 1.1251 |
| *CSNK1A1* | 1.1975 | 1.6245 | 1.84037 | 2.46228 | 3.0738 |
| *CSNK1D* | 2.2815 | 2.2815 | 1.4539 | 1.5801 | 1.8277 |
| *CSNK1G1* | 1.4743 | -1.97246 | 1.414214 | 1.505247 | 1.239708 |
| *CSNK2A1* | -1.5052 | 1.454 | 1.366 | -1.1173 | -1.2924 |
| *CTBP1* | 1.86606 | 2.6207 | 2.92817 | 3.5308 | 3.3869 |
| *CTBP2* | 1.3195 | 1.1019 | 2.25011 | 2.4283 | 1.95884 |
| *CTNNB1* | 1.58008 | 1.84037 | 2.2974 | 2 | 2.09943 |
| *CTNNBIP1* | 1.474269 | 2.411616 | 2.907945 | 1.753211 | 4.40762 |
| *CXXC4* | -1.2311 | -2.2721 | -1.8609 | -1.1777 | 1.1329 |
| *DAAM1* | -1.2058 | 1.3104 | 1.2658 | 2.9282 | 1 |
| *DIXDC1* | -1.59107 | -1.3947 | -5.2054 | -1.9185 | 1.1892 |
| *DKK1* | -4.2871 | -2.3198 | 1.031 | 1.3698 | 1.3379 |
| *DVL1* | 1.474269 | 1.172835 | 2.084932 | 4.69134 | 1.375542 |
| *DVL2* | 1.693491 | 1.385109 | 1.569168 | 1.049717 | 2.770219 |
| *EP300* | 2.0705 | 1.52625 | 2.1199 | 3.42 | 1.9185 |
| *FBXW11* | -1.9725 | 1.2108 | 1.1942 | 1.5094 | 1.2834 |
| *FBXW2* | -1.257 | 1.3435 | 1.3435 | 1.4682 | 1.1251 |
| *FGF4* | 1.0718 | -1.2746 | -3.2944 | -1.5369 | 1.0718 |
| *FOSL1* | -1.4439 | -1.5801 | -3.4105 | -2.5315 | -1.5583 |
| *FOXN1* | 1 | 1 | 1 | 1 | 1.6935 |
| *FRAT1* | -1.1408 | -1.0425 | -1.366 | -1.366 | -1.0792 |
| *FRZB* | -5.0281 | -3.3265 | -2.4726 | -1.967 | -1.37 |
| *FSHB* | -1.014 | -1.0792 | 1 | 1 | -1.5476 |
| *FZD1* | -1.6935 | 1.4241 | -1.1173 | -1.3013 | -1.1329 |
| *FZD2* | 1.1096 | -1.3287 | -1.7053 | 1.3851 | 2.362 |
| *FZD6* | -1.0281 | 1.6702 | 1.057 | -1.7901 | 1.4241 |
| *FZD7* | -1.8025 | 1.1975 | 4.4691 | -1.4044 | 1.2746 |
| *FZD8* | -1.0497 | 1.3755 | -1.9588 | 1.4241 | 1.1329 |
| *GSK3A* | 1.007 | 2.3457 | 1.007 | -1.1567 | 1.454 |
| *GSK3B* | -1.181 | 1.3947 | -1.4743 | 1.1329 | 1.7291 |
| *JUN* | 1.0718 | 1.2888 | 2.3685 | 2.5036 | 3.0738 |
| *KREMEN1* | -1.1096 | -1.0497 | -1.1173 | 1.3013 | 1.3472 |
| *LEF1* | 1.11728 | -1.47426 | 2.2501 | 1.879 | 1.4743 |
| *LRP5* | -1.3195 | -1.815 | 1.5583 | 1.3755 | 1.8025 |
| *LRP6* | 1.366 | -1.1892 | 1.434 | 1.6358 | 1.3195 |
| *MYC* | 1.24833 | 1.15668 | 1.2008 | 1.5094 | 2.9079 |
| *NKD1* | 1.4948 | 1.014 | 1.257 | 1.9185 | 1.7053 |
| *NLK* | -1.4439 | 1.0614 | 1.1219 | 1.5735 | 1.2142 |
| *PITX2* | -1.0353 | 1.7777 | -1.6021 | 1.014 | 1.2658 |
| *PORCN* | 1.164734 | 1.658639 | 1.624505 | 1.42405 | 0.933033 |
| *PPP2CA* | 2.462289 | 1.049717 | 0.993092 | 1.972465 | 2.12874 |
| *PPP2R1A* | -1.1173 | 1.5977 | 1.5094 | 1.5305 | 1.021 |
| *PYGO1* | 1.0943 | 1.5692 | 1.3472 | 1.0353 | 1.2226 |
| *RHOU* | -1.454 | -1.0281 | -1.5801 | 1.057 | 1.4743 |
| *SENP2* | 8.515 | 1.2142 | 3.5308 | 3.8906 | 1.5369 |
| *SFRP1* | 1.1567 | -1.1251 | 2.5847 | 2.3457 | 1.021 |
| *SFRP4* | -1.1728 | -5.2561 | -2.5955 | -1.81 | 2.3134 |
| *FBXW4* | 1.8921 | 1.0762 | 1.2008 | 2.7587 | 1.9588 |
| *SLC9A3R1* | 2.027919 | 1.765406 | 3.226567 | 2.34567 | 1.681793 |
| *T* | 1.4142 | 1.6132 | 1.4742 | 1.58 | 2.042 |
| *TCF7* | 2.114 | 1.3909 | 1.168 | 1.629 | 1.6245 |
| *TCF7L1* | -1.6702 | 1.2311 | -1.2058 | -1.1487 | -1.3195 |
| *TLE1* | 1.777685 | 1.101905 | 2.907945 | 2.462289 | 1.613284 |
| *TLE2* | -1.591 | -2.5315 | -1.3195 | -1.6472 | -1.1728 |
| *WIF1* | 1.292353 | 0.946058 | 3.5801 | 3.506423 | 1.681793 |
| *WNT1* | 1.0497 | 1 | 1 | 1 | 1.1487 |
| *WNT2* | -1.1329 | -1.4948 | -1.7053 | -2.2345 | -1.1728 |
| *WNT2B* | -1.31951 | -1.16473 | 1.049717 | 1.301342 | -1.25701 |
| *WNT3* | 1.3755 | 1.6818 | -1.434 | -1.0497 | 1.1019 |
| *WNT3A* | -1.366 | -1.1975 | -2.0562 | 1.4142 | 1.4948 |
| *WNT4* | -1.32869 | -2.07052 | -1.11729 | -2.1287 | -2.04202 |
| *WNT5A* | -1.4241 | 1.4948 | 1.3755 | 1.3379 | 1.1567 |

^a^ Numbers represent the fold-change in the expression levels of genes relative to their levels in unstimulated, serum-starved cells. The data are the means of three biological replicates. Only genes whose mean transcript levels changed by more than two-fold at one or more time points were considered significant. Positive numbers represent upregulation; negative numbers represent downregulation.
